# HLA-C*04 and hepatocellular carcinoma post-surgical survival in Hawaii

**DOI:** 10.3389/fgene.2026.1874308

**Published:** 2026-10-26

**Authors:** Sarah Abdul-Ghani, Noa Brenner, Brenda Y. Hernandez, Linda Wong, Hyeong Jun Ahn, Benjamin L. Green

**Affiliations:** 1 Cancer Biology Program, University of Hawai’i Cancer Center, Honolulu, HI, United States; 2 Department of Quantitative Health Sciences, John A. Burns School of Medicine, University of Hawaii at Manoa, Honolulu, HI, United States; 3 Population Sciences in the Pacific, University of Hawai’i Cancer Center, Honolulu, HI, United States; 4 Department of Surgery, Queen’s Medical Center, Honolulu, HI, United States

**Keywords:** Hawaii, HCC, HLA-C*04, MELD, transplant

## Abstract

**Background:**

The role of human leukocyte antigen (HLA) allotype in hepatocellular carcinoma (HCC)-specific outcomes is poorly understood.

**Methods:**

We performed a retrospective analysis of patients with HCC treated surgically in Hawaii at a single, tertiary care institution. HLA allotyping was performed as clinically indicated for patients undergoing orthotopic liver transplantation (OLT), and commercial HLA genotyping was performed on PBMCs collected from patients who underwent partial hepatectomy (PH). The relationship of HLA with overall survival (OS) was evaluated by univariable and multivariable Cox proportional hazards models.

**Results:**

From 2012 to 2024, 84 patients underwent OLT with HLA assessment (n = 43) or PH with banked PBMCs available for genotyping (n = 41). The median age was 62; there were 20% female patients; and 50% and 10% identified as Asian and Pacific Islander, respectively. Nearly 70% were BCLC 0 or A, and the median tumor size was 3 cm. HCC etiology was 19% NAFLD, 38% HBV, and 42% HCV. The multivariable model identified a history of non-HCC malignancies, tumor size, MELD score, and the presence of at least one germline allele of HLA-C*04 to be significantly correlated with worse postoperative OS (*p* < 0.05).

**Conclusion:**

Among an ethnically diverse population of mostly early-stage patients treated with OLT or PH in Hawaii, exploratory analysis showed that disease-specific factors and HLA-C*04 allotype were independently associated with worse postoperative OS. Based on this hypothesis-generating study, future studies should evaluate whether the unique properties of Hawaii’s HCC surgical population include an immuno-genomic mechanism affecting outcome.

## Introduction

Hepatocellular carcinoma (HCC) represents a substantial global burden of disease as the sixth most commonly diagnosed cancer and third most common cause of cancer death worldwide (Bray et al., 2024). HCC is endemic in Pacific Islander and Asian populations (Mak et al., 2024). Systemic and locoregional therapies for late-stage disease have improved, but low rates of cure necessitate continued study of HCC biology. When detected early enough, a minority of cases are candidates for surgical treatment (transplant or resection) (Malik et al., 2025). Despite curative intent surgery, recurrence is common. Preoperative factors that impact patient survival following surgical management of HCC are largely unknown.

Human leukocyte antigen (HLA) molecules present immunogenic peptides to activate T cells and drive an antigen-specific adaptive immune response (Choo, 2007). Encoded by specific genotypes, certain HLA allotypes have been associated with disease prevalence and therapy response for immunological processes (Gao et al., 2019; Chowell et al., 2018). Furthermore, HLAs are a source of immense genetic diversity. Various HLA allotypes exhibit ethno-geographic clustering, making HLA-outcome correlations potentially unique to certain local populations (Arrieta-Bolaños et al., 2023). Finally, HLA allotyping is a routine clinical assay during the workup of patients under consideration for liver transplantation, including those candidates with early-stage HCC as defined by the Milan criteria. The impact of HLA allotypes on early-stage HCC generally, and in Hawaii specifically, is unknown (Kawam et al., 2023; Singh et al., 2007). The purpose of this study was to determine whether survival among surgical patients with HCC correlated with differences in HLA allotype.

## Methods

### Inclusion criteria and endpoints

A prospectively-maintained single-institution database was queried. Patients at least 18 years of age with histologically confirmed HCC who underwent orthotopic liver transplantation (OLT) or partial hepatectomy (PH) between 2012 and 2024 were included. Patients were excluded if they did not undergo surgery or had no HLA data. Demographic and clinico-pathologic variables were collected. The primary endpoint was postoperative overall survival (OS) defined as the time period between surgery and death or last contact. A complete case analysis (patients only with non-missing data for all variables) was used in multivariable Cox-based analyses. For descriptive analyses and univariate Cox-based analyses, all available data was utilized per variable. All participants provided written informed consent for the study, which was approved by an Institutional Review Board.

### HLA allotyping

For pre-operative blood samples from patients who underwent PH, DNA was extracted from buffy coats using QIamp DNA Blood Mini extraction kit (Qiagen, Germantown, MD, USA). HLA typing was analyzed using AlloSeq Tx 17.1 library prep kit (CareDx, Brisbane CA, USA) following the manufacturer’s protocol. Obtained fastq files were used as an input to AlloSeq Assign software. Allotypes were determined from genotyping data using an electronic database (https://www.ebi.ac.uk/ipd/imgt/hla/, accessed 01/2025).

HLA allotype data was collected for OLT patients through the existing database, as it was performed as part of standard pre-transplant workup. Patients with missing follow up data or dates of treatment were removed from the study.

### Statistical analysis

Baseline demographic and clinicopathological patient characteristics were summarized using descriptive statistics and stratified by HLA-C*04 status. Categorical measures were displayed as counts and frequencies, while medians and interquartile ranges were calculated for continuous variables. Pearson’s chi-squared tests and Fisher’s exact tests were used to measure statistical significance for categorical variables, depending on sample sizes. Wilcoxon rank sum tests were used to measure statistical significance for continuous variables.

OS was evaluated using Kaplan-Meier survival analysis, with differences in survival curves assessed using the log-rank test. Kaplan-Meier analyses were performed overall according to HLA-C*04 status and also by treatment modality (PH and OLT). Firth’s penalized univariate and multivariable Cox proportional hazards regression analyses were conducted to evaluate the association between clinicopathological variables and HLA variables with OS. Variables with a p-value < 0.10 in univariate analysis or established clinical relevance were included in the multivariable Cox regression model.

To address potential multicollinearity, variance inflation factors (VIFs) were calculated for all candidate predictors. Variables with a VIF > 5 were excluded from the final model to ensure interpretability and stability (Dormann et al., 2013). Additionally, the proportional hazards assumption was tested using Schoenfeld residuals using a standard Cox model corresponding to each Firth-penalized fit. Any predictors violating this assumption were removed. The final multivariable Cox model was then used to interpret true independent associations between selected predictors and survival.

Additional analyses were performed to further evaluate the association between HLA-C*04 and survival. The interaction between HLA-C*04 status and treatment modality was assessed using univariate and multivariable Firth-penalized Cox proportional hazards models including an interaction term. A sensitivity analysis was also performed by repeating the Firth-penalized Cox analyses after excluding patients homozygous for HLA-C*04. To account for multiple hypothesis testing, Benjamini-Hochberg false discovery rate-adjusted p-values were calculated for the univariate and multivariable Firth-penalized Cox regression analyses.

Firth’s penalized Cox proportional hazards models were generated using the *survival* R package (Singh et al., 2007). KM curves were visualized using the *survminer* R package. Statistical significance was set to p < 0.05 and all programming was conducted in R version 4.4.1.

## Results

### Patient and disease characteristics

From 2012 to 2024, 43 patients (51%) underwent OLT for HCC (Table 1). An additional 41 patients that underwent PH and had biobanked PBMCs were included in the study for a total of 84 patients, all of whom had HLA and clinical follow up data. There were 17 females and 67 males, with a median age of 62. Patient self-identified ethnicity included 42 Asian (50%), 8 Pacific Islander (10%), and 16 Caucasian (19%). There were substantial HCC-related co-morbidities in the cohort, including HBV (n = 32, 38%), HCV (n = 35, 42%), a history of alcohol use (n = 31, 37%), and diagnosis of NASH/NAFLD (n = 16, 19%). The median BMI was 27. Other co-morbid conditions present in the population were a history of smoking (n = 53, 63%), diabetes (n = 35, 42%), hyperlipidemia (n = 31, 37%), and hypertension (n = 45, 54%). Other (non-HCC) malignancies were found in 12 patients (14%).

**TABLE 1:** Clinico-pathologic variables.

Patient-specific variables (n, %)	n	%
Age (years, median)	62	​
Female	17	20%
Pacific Islander	8	10%
Asian	42	50%
Caucasian	16	19%
Mixed	13	15%
Black or Puerto Rican	4	5%
Co-morbidities (n, %)		
HBV	32	38%
HCV	35	42%
Alcohol use	31	37%
NASH/NAFLD	16	19%
Other malignancies	12	14%
BMI (median)	27	​
Smoking history	53	63%
Diabetes	35	42%
Hyperlipidemia	31	37%
Hypertension	45	54%
Disease-specific variables (n, %)		
Largest tumor size (cm, median)	3	​
Largest tumor over 3 cm	45	54%
More than 1 tumor	17	20%
Cirrhosis	55	65%
MELD at diagnosis (median)	8	​
AFP at diagnosis (ng/mL, median)	9.5	​
Transplant	43	51%
Resection	41	49%
Post-operative survival (median days)	1,667	​
Post-diagnosis survival (median days)	1,876	​
BCLC		
0	7	9%
A	3	4%
A1	24	30%
A2	9	11%
A3	7	9%
A4	5	6%
B	17	21%
C	2	2%
D	7	9%

At the time of diagnosis, the median tumor size was 3 cm (Table 1). Of the 81 patients with BCLC staging, the majority were early stage, with 68% classified as either BCLC-0 or BCLC-A. There were 45 patients (54%) that had at least one HCC tumor over 3 cm, and 17 patients (20%) that had more than one tumor. Cirrhosis was present in 55 patients (65%). The median MELD score at diagnosis was 8; the median serum AFP at diagnosis was 9.5 ng/mL. Median overall survival for the cohort was 1,667 days postoperatively and 1876 days post-diagnosis, and there were 18 total deaths during the follow up period.

### Uni- and multivariable survival models

First, we sought to determine the impact of each clinicopathological variable on postoperative survival. Firth penalized univariate Cox proportional hazards analysis was performed. Reduced OS was significantly associated with a history of non-HCC primary cancer (HR = 3.68, 95%CI 1.41–9.58, *p* = 0.008) and MELD score (HR = 1.12, 95%CI 1.01–1.25, *p* = 0.040), while a history of alcohol use (*p =* 0.057) and larger tumors (*p* = 0.053) were approaching significance (Table 2). Sex, age, BCLC stage, type of surgery (OLT vs. PH) and other comorbidities were not significantly associated with postoperative survival (Figure 1).

**TABLE 2:** Firth’s penalized univariate and multivariable Cox proportional hazards analyses.

Univariate analysis		
Predictor	HR (95% CI)	p-value
Sex	0.87 (0.26, 2.89)	0.82
Age	1.03 (0.97, 1.09)	0.385
Orthotopic liver transplant	0.56 (0.19, 1.65)	0.293
Other malignancies	3.68 (1.41, 9.58)	**0.008**
Largest tumor >3 cm	2.75 (0.99, 7.69)	0.053
Cirrhosis	0.37 (0.14, 0.97)	**0.043**
MELD	1.12 (1.01, 1.25)	**0.04**
NASH/NAFLD	2.11 (0.75, 5.94)	0.157
Alcohol use	2.49 (0.97, 6.37)	0.057
Smoking history	2.18 (0.73, 6.51)	0.163
HLA-A*02	1.18 (0.46, 3.04)	0.731
HLA-A*24	1.99 (0.75, 5.28)	0.168
HLA-B*35	1.29 (0.46, 3.60)	0.624
HLA-BW*04	1.30 (0.49, 3.46)	0.597
HLA-C*04	2.62 (0.97, 7.08)	0.058
HLA-C*09	2.96 (0.90, 9.76)	0.075
HLA-DR*11	3.21 (0.89, 11.53)	0.074
HLA-DR*12	2.18 (0.73, 6.53)	0.165
HLA-DR*15	1.16 (0.42, 3.21)	0.776
HLA-DR*51	1.10 (0.41, 2.90)	0.855
HLA-DR*52	1.77 (0.67, 4.70)	0.25
HLA-DR*53	0.68 (0.26, 1.75)	0.424
HLA-DQ*05	0.89 (0.35, 2.26)	0.81
HLA-DQ*06	1.29 (0.50, 3.34)	0.593
HLA-DQ*07	2.37 (0.92, 6.12)	0.074

Multivariable analysis		
Predictor	HR (95% CI)	p-value
Female (vs. male)	2.53 (0.42, 15.31)	0.312
Treatment: OLT	0.75 (0.10, 5.83)	0.786
MELD	1.24 (1.06, 1.46)	**0.009**
Cirrhosis	0.32 (0.07, 1.51)	0.150
Other malignancies	5.72 (1.34, 24.42)	**0.019**
Largest tumor over 3 cm	5.34 (1.32, 21.65)	**0.019**
Alcohol use	2.17 (0.55, 8.60)	0.272
HLA-C*04	4.08 (1.02, 16.29)	**0.047**
HLA-C*09	3.47 (0.65, 18.40)	0.144
HLA-DR*11	4.29 (0.54, 33.88)	0.167
HLA-DQ*07	0.65 (0.15, 2.87)	0.567
Hyperlipidemia	0.92 (0.20, 4.27)	0.918

Bold indicates p < 0.05.

**FIGURE 1:**
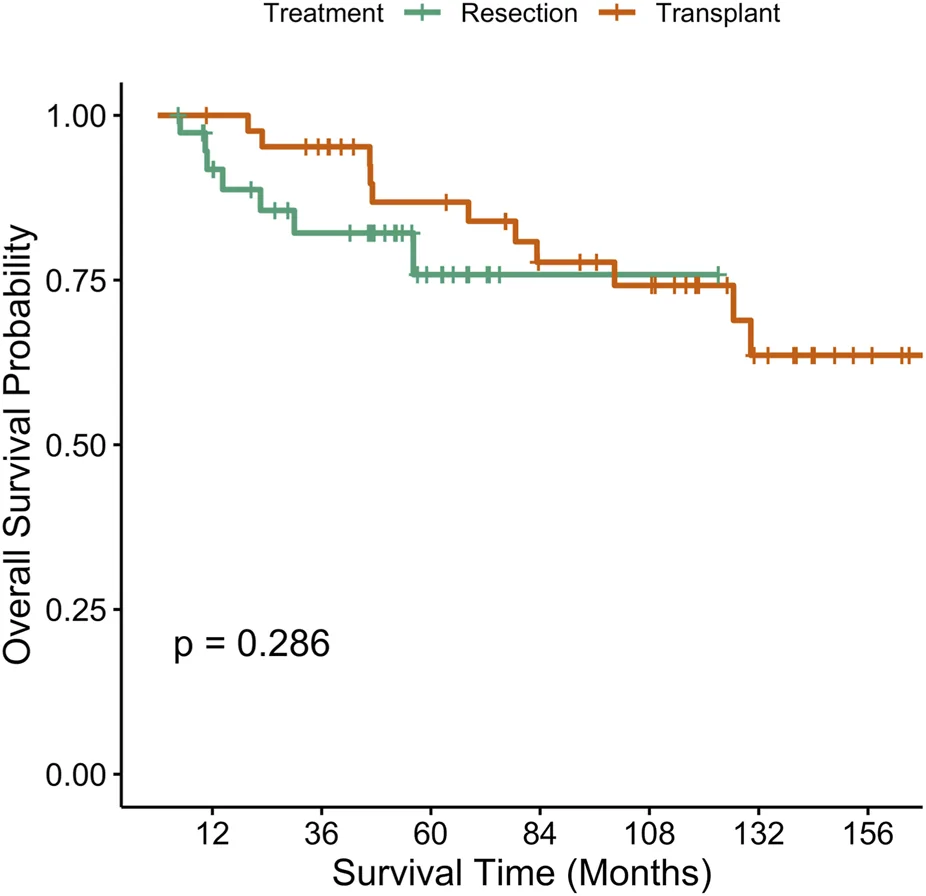
Survival analysis of patients that underwent PH compared with OLT.

All patients that underwent OLT had routine clinical HLA testing, and PH patients had HLA allotype determined through analysis of PBMC genotyping. Patients were considered positive for an HLA allotype if they tested positive for at least one copy of the allele. No HLA allotype was found to be significantly associated with decreased survival, although HLA-C*04 (*p* = 0.058) approached significance (Figure 2; Table 2).

**FIGURE 2:**
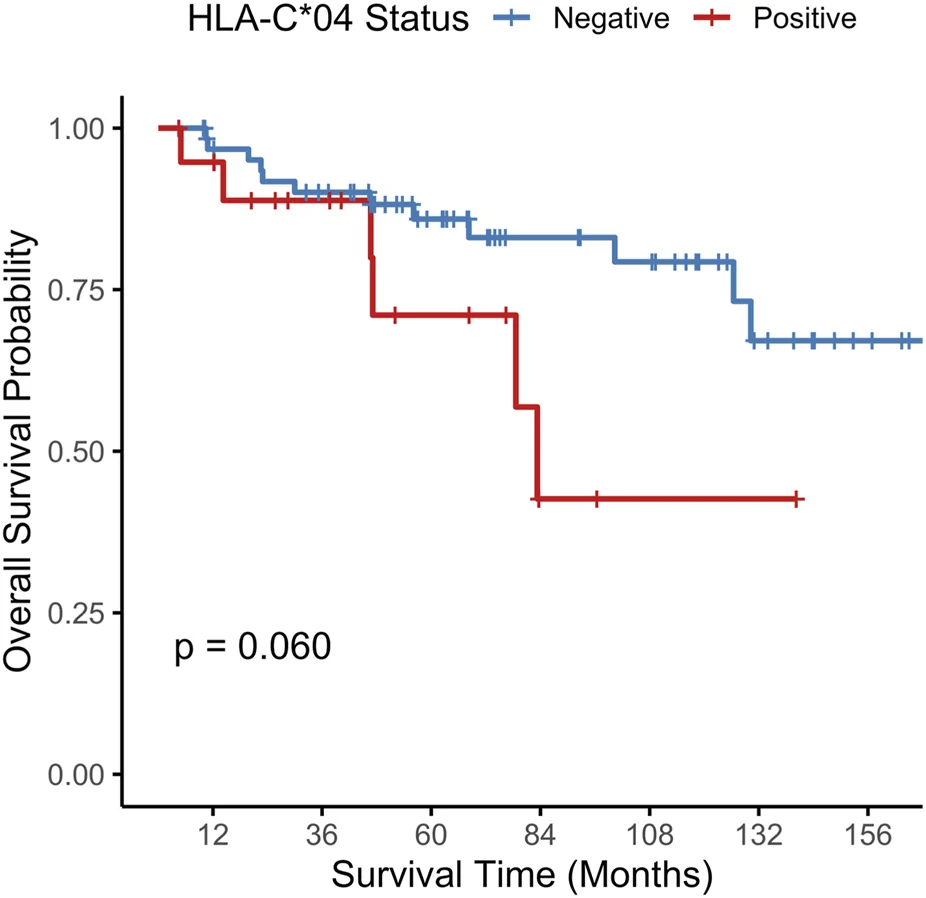
Survival analysis of patients that were positive for the HLA-C*04 allotype compared with HLA-C*04-negative.

Variables reaching a significance level of *p* = 0.10 for a decrease in OS on univariable analysis or selected based on clinical relevance (i.e., treatment type, hyperlipidemia) were collected into a separate multivariable model. In this model, increasing MELD score (HR = 1.24, 95%CI 1.06–1.46, *p* = 0.009), a history of non-HCC malignancy (HR = 5.72, 95%CI 1.34–24.42, *p* = 0.019), largest tumor diameter over 3 cm (HR = 5.34, 95%CI 1.32–21.65, *p* = 0.019), and at least one locus of the allotype HLA-C*04 (HR = 4.08, 95%CI 1.02–16.29, *p* = 0.047) were independently associated with reduced postoperative OS (Table 2). No other HLA alleles included in the multivariable model were significantly associated with changes in OS.

### HLA-C*04 subgroup analysis

Further analysis was performed on the patients positive for the HLA-C*04 allotype, summarized in Table 3. Of the 20 patients in this subgroup, there were 10 OLT and 10 PH patients. Three patients were homozygous for HLA-C*04. There were 6 deaths in the HLA-C*04 subgroup at the end of follow up. Similar values between the entire cohort and the HLA-C*04 subgroup were seen for age (median years 62 vs. 63), Asian ethnicity (50% vs. 55%), and Caucasian ethnicity (19% vs. 15%). Of the 8 total Pacific Islanders in the cohort (10%), 4 were positive for HLA-C*04. The HLA-C*04 subgroup had lower rates of HCV (25% vs. 42%, ns) and >1 HCC tumor (5% vs. 20%, ns) compared with the larger cohort. However, the frequency of NASH/NAFLD (35% vs. 19%, ns), hypertension (70% vs. 54%, ns), and hyperlipidemia (65% vs. 37%, p = 0.001) were all higher in the HLA-C*04 subgroup compared with the larger cohort, despite a similar median BMI (27 vs. 25).

**TABLE 3:** Descriptive Statistics stratified by HLA-C*04.

Characteristic	Overall, N = 84^a^	Negative (−), N = 64^a^	Positive (+), N = 20^a^	p-value^b^
Age median, (range)	62.00 (56.75, 67.00)	62.00 (57.00, 67.00)	63.00 (51.75, 67.50)	0.8
Sex	​	​	​	0.2
Male	67 (80%)	53 (83%)	14 (70%)	​
Female	17 (20%)	11 (17%)	6 (30%)	​
Pacific Islander	​	​	​	0.087
Not Pacific Islander	76 (90%)	60 (94%)	16 (80%)	​
Pacific Islander	8 (9.5%)	4 (6.2%)	4 (20%)	​
Asian	​	​	​	0.6
Not asian	42 (50%)	33 (52%)	9 (45%)	​
Asian	42 (50%)	31 (48%)	11 (55%)	​
Caucasian	​	​	​	0.8
Not caucasian	68 (81%)	51 (80%)	17 (85%)	​
Caucasian	16 (19%)	13 (20%)	3 (15%)	​
Mixed race	​	​	​	0.2
Not mixed race	71 (85%)	52 (81%)	19 (95%)	​
Mixed race	13 (15%)	12 (19%)	1 (5.0%)	​
Black or Puerto Rican	​	​	​	0.6
Not Black or Puerto Rican	80 (95%)	60 (94%)	20 (100%)	​
Black or Puerto Rican	4 (4.8%)	4 (6.2%)	0 (0%)	​
Alcohol use	​	​	​	0.8
No alcohol usage	53 (63%)	40 (62%)	13 (65%)	​
Alcohol usage	31 (37%)	24 (38%)	7 (35%)	​
NASH/NAFLD	​	​	​	0.052
No NASH/NAFLD	68 (81%)	55 (86%)	13 (65%)	​
NASH/NAFLD	16 (19%)	9 (14%)	7 (35%)	​
Other malignancies	​	​	​	>0.9
No other malignancies	72 (86%)	55 (86%)	17 (85%)	​
Other malignancies	12 (14%)	9 (14%)	3 (15%)	​
BMI, median (range)	26.65 (23.10, 29.70)	26.85 (23.08, 29.47)	25.10 (24.03, 30.27)	0.9
Smoking history	​	​	​	0.4
No smoking history	31 (37%)	22 (34%)	9 (45%)	​
Smoking history	53 (63%)	42 (66%)	11 (55%)	​
Diabetes	​	​	​	0.9
No diabetes	49 (58%)	37 (58%)	12 (60%)	​
Diabetes	35 (42%)	27 (42%)	8 (40%)	​
Hyperlipidemia	​	​	​	**0.003**
No hyperlipidemia	53 (63%)	46 (72%)	7 (35%)	​
Hyperlipidemia	31 (37%)	18 (28%)	13 (65%)	​
Hypertension	​	​	​	0.1
No hypertension	38 (46%)	32 (51%)	6 (30%)	​
Hypertension	45 (54%)	31 (49%)	14 (70%)	​
Largest tumor size, cm (range)	3.15 (2.30, 4.43)	3.00 (2.28, 4.40)	3.75 (2.30, 4.62)	0.5
Largest tumor >3 cm	​	​	​	0.5
No tumor over 3 cm	39 (46%)	31 (48%)	8 (40%)	​
Tumor over 3 cm	45 (54%)	33 (52%)	12 (60%)	​
More than 1 tumor	​	​	​	0.061
No more than 1 tumor	67 (80%)	48 (75%)	19 (95%)	​
More than 1 tumor	17 (20%)	16 (25%)	1 (5.0%)	​
MELD score median (range)	8.00 (7.00, 12.00)	8.00 (6.75, 12.00)	9.00 (7.00, 11.50)	0.5
AFP ng/dL (range)	9.50 (5.07, 85.28)	8.35 (5.27, 94.40)	11.10 (3.13, 30.00)	0.4
Cirrhosis	​	​	​	0.3
No liver cirrhosis	29 (35%)	20 (31%)	9 (45%)	​
Liver cirrhosis	55 (65%)	44 (69%)	11 (55%)	​
Transplant	​	​	​	>0.9
No transplant	41 (49%)	31 (48%)	10 (50%)	​
Had transplant	43 (51%)	33 (52%)	10 (50%)	​
Resection	​	​	​	0.7
No resection	45 (54%)	35 (55%)	10 (50%)	​
Had resection	39 (46%)	29 (45%)	10 (50%)	​
Vital status	​	​	​	0.4
Deceased	66 (79%)	52 (81%)	14 (70%)	​
Alive	18 (21%)	12 (19%)	6 (30%)	​

^a^ Median (IQR); n (%).

^b^ Wilcoxon rank sum test; Fisher’s exact test; Pearson’s Chi-squared test.

Bold indicates p < 0.05.

## Discussion

In this study, we pooled data from patients with HCC undergoing OLT and PH to examine potential associations of clinicopathological characteristics and post-operative OS. Similar to previous studies of surgically treated HCC (Sarkar et al., 2017; Hong et al., 2015; Mazzaferro et al., 1996), tumor size, secondary cancers, and MELD score were independently and inversely associated with postoperative OS. In addition, our cohort identified the HLA-C*04 allotype as an independently negative prognostic variable for postoperative OS.

Candidates for OLT falling within Milan criteria constitute a small but important subgroup of all patients with HCC, whose early disease confers a favorable prognosis with curative surgical management. Prior to transplant, recipient HLA allotypes are routinely obtained to optimize graft compatibility due to their property as a natural source of allogenic diversity capable of mediating rejection (Choo, 2007). We leveraged HLA allotyping performed preoperatively for OLT as curative therapy for HCC to test the hypothesis that certain HLA allotypes confer survival differences in HCC. To accommodate the numerous subcategories and low statistical power derived from subsetting patients by HLA allotype, we (1) added a secondary cohort of patients that had undergone PH for HCC and had both survival outcomes and biobanked PBMCs, (2) constrained our analysis to classifying patients by the presence of one copy of the HLA allele, (3) used Firth’s penalized Cox proportional hazards models for statistical analysis.

Certain HLA allotypes (Chowell et al., 2018) and diversity (Chowell et al., 2019) are associated with survival in solid cancers, although the effect on immunotherapy response is debated (Chhibber et al., 2022). HLA-C*04 is present in certain Asian populations (Dunn et al., 2016; Que et al., 2022), but studies on its immunologic properties are limited (Weiner et al., 2021). The HLA-C*04:01 genotype is among the most highly expressed of any HLA alleles in normal breast and lung tissue (Puttick et al., 2024). HLA-C*04 is capable of loading the GPC3 tumor-associated peptide and inducing an antigen-specific T cell reaction (Vercher et al., 2025). Although patients with a germline HLA-C*04 copy had reduced survival in a cohort with HNSCC (Dyckhoff et al., 2022), no group has previously linked the allele to HCC outcomes. In our cohort, the 20 patients with at least one copy of HLA-C*04 had higher rates of hyperlipidemia, hypertension, and NASH/NAFLD, but lower rates of HCV and multiple HCC tumors. No other HLA allotypes were significantly associated with changes in postoperative OS.

The surgical HCC population consists of a unique combination of disease features that includes favorable anatomy and patient physiology conducive to resection. Accordingly, our cohort had a relatively long survival compared with historical cohorts of non-surgical candidates, with a median overall survival of 1,668 days including multiple long-term (>10 years) survivors. Patient-specific variables such as the type of resection (OLT vs. PH), medical history (smoking history, race, disease etiology, BMI), AFP, and BCLC scores did not correlate with survival. As survival timepoints were standardized to date of operation, and differences only arose after prolonged follow up beyond what is typical in studies of advanced HCC, our findings may not be applicable to non-surgical candidates. Future work should confirm whether HLA allotypes correlate with survival outside of early stage, predominantly Asian and Pacific Islander populations.

There were other limitations to this retrospective study. The inclusion of numerous HLA allotypes in the analysis was exploratory in nature and, combined with a sample size limited to our institutional experience, the conclusions should be considered hypothesis-generating rather than definitive. There was risk of false discovery, as a Multiple Testing Correction (Benjamini-Hochberg method) performed did not demonstrate any variable as statistically associated with OS, including HLA-C*04 positivity (Supplementary Table S1). While we did include all variables from the univariable model showing *p* ≤ 0.10 into the multivariable model, inclusion of three metabolic variables that were higher at this threshold in the HLA*C-04 group (i.e., hypertension, hyperlipidemia, NASH) generated model instability; only hyperlipidemia was selected for the multivariable model due to its significance threshold *p* ≤ 0.05. Although OLT/PH and treatment type did not affect the association between HLA-C*04 and OS on interaction analysis (Supplementary Table S2), half of our cohort was immunocompromised post-OLT, potentially driving differential effects of HLA on the immune control of HCC between the two groups. In addition, HLA diversity resulted in low statistical power in many of the allotype groups. To account for this limitation, the presence of at least one copy of an HLA allotype, not HLA combinations, genotypes, or homozygosity, were considered for patient grouping. Our study did not detect the protein-level expression of any HLA, which is known to decrease as a mechanism of immune escape in HCC (Shigematsu et al., 2023). Finally, OS over a long-term follow up may not accurately reflect the immune-mediated disease control mechanism; future studies may benefit from selecting RFS or HCC-specific survival as an endpoint to isolate the HLA-specific contribution.

In summary, we identified HLA-C*04 as a putative negative prognostic marker postoperatively for patients with HCC in Hawaii. Understanding of the immuno-genomic contribution to survival may enable a more personalized discussion of risks/benefits of surgery, as well as empower next-generation immunotherapy development.

## Data Availability

The datasets generated and/or analyzed during the current study are not publicly available due to institutional privacy restrictions but are available from the corresponding author upon reasonable request.
