# Supplementary material for: HLA-C*04 and hepatocellular carcinoma post-surgical survival in Hawaii
Source: Front Genet. 2026 Oct 26;17:1874308. doi: 10.3389/fgene.2026.1874308 (PMC13745682; doi:10.3389/fgene.2026.1874308)
Supplement: Supplementary file 1 [file Table1.docx]

## SUPPLEMENTARY TABLE 1: Multiple Testing Correction (Benjamini-Hochberg FDR)

### Univariate Model

Univariate Firth-Penalized Cox Models

| Predictor | HR (95% CI) | p-value | FDR p-value |
| --- | --- | --- | --- |
| Sex | 0.87 (0.26, 2.89) | 0.820 | 0.854 |
| Age | 1.03 (0.97, 1.09) | 0.385 | 0.602 |
| Treatment | 0.56 (0.19, 1.65) | 0.293 | 0.488 |
| Other malignancies | 3.68 (1.41, 9.58) | **0.008** | 0.191 |
| Largest tumor >3 cm | 2.75 (0.99, 7.69) | 0.053 | 0.208 |
| Cirrhosis | 0.37 (0.14, 0.97) | **0.043** | 0.208 |
| MELD | 1.12 (1.01, 1.25) | **0.040** | 0.208 |
| NASH/NAFLD | 2.11 (0.75, 5.94) | 0.157 | 0.322 |
| Alcohol use | 2.49 (0.97, 6.37) | 0.057 | 0.208 |
| Smoking history | 2.18 (0.73, 6.51) | 0.163 | 0.322 |
| HLA-A*02 | 1.18 (0.46, 3.04) | 0.731 | 0.854 |
| HLA-A*24 | 1.99 (0.75, 5.28) | 0.168 | 0.322 |
| HLA-B*35 | 1.29 (0.46, 3.60) | 0.624 | 0.780 |
| HLA-BW*04 | 1.30 (0.49, 3.46) | 0.597 | 0.780 |
| HLA-C*04 | 2.62 (0.97, 7.08) | 0.058 | 0.208 |
| HLA-C*09 | 2.96 (0.90, 9.76) | 0.075 | 0.208 |
| HLA-DR*11 | 3.21 (0.89, 11.53) | 0.074 | 0.208 |
| HLA-DR*12 | 2.18 (0.73, 6.53) | 0.165 | 0.322 |
| HLA-DR*15 | 1.16 (0.42, 3.21) | 0.776 | 0.854 |
| HLA-DR*51 | 1.10 (0.41, 2.90) | 0.855 | 0.855 |
| HLA-DR*52 | 1.77 (0.67, 4.70) | 0.250 | 0.446 |
| HLA-DR*53 | 0.68 (0.26, 1.75) | 0.424 | 0.623 |
| HLA-DQ*05 | 0.89 (0.35, 2.26) | 0.810 | 0.854 |
| HLA-DQ*06 | 1.29 (0.50, 3.34) | 0.593 | 0.780 |
| HLA-DQ*07 | 2.37 (0.92, 6.12) | 0.074 | 0.208 |

### Multivariable Model

Multivariable Firth Penalized Cox Model

| Predictor | HR (95% CI) | p-value | FDR p-value |
| --- | --- | --- | --- |
| Age (y) | 1.01 (0.93, 1.09) | 0.884 | 0.918 |
| Female (vs Male) | 2.53 (0.42, 15.31) | 0.312 | 0.451 |
| Treatment | 0.75 (0.10, 5.83) | 0.786 | 0.918 |
| MELD | 1.24 (1.06, 1.46) | **0.009** | 0.082 |
| Cirrhosis | 0.32 (0.07, 1.51) | 0.150 | 0.311 |
| Other malignancies | 5.72 (1.34, 24.42) | **0.019** | 0.082 |
| Largest Tumor Over 3cm | 5.34 (1.32, 21.65) | **0.019** | 0.082 |
| Alcohol Use | 2.17 (0.55, 8.60) | 0.272 | 0.442 |
| HLA-C*04 | 4.08 (1.02, 16.29) | **0.047** | 0.152 |
| HLA-C*09 | 3.47 (0.65, 18.40) | 0.144 | 0.311 |
| HLA-DR*11 | 4.29 (0.54, 33.88) | 0.167 | 0.311 |
| HLA-DQ*07 | 0.65 (0.15, 2.87) | 0.567 | 0.737 |
| Hyperlipidemia | 0.92 (0.20, 4.27) | 0.918 | 0.918 |

## SUPPLEMENTARY TABLE 2: Univariate and Multivariable Cox Analysis with Interaction between HLA-C*04 and Treatment Type

Unadjusted Firth Penalized Cox Model

| Predictor | HR (95% CI) | p-value |
| --- | --- | --- |
| HLA-C*04 | 2.59 (0.51, 13.11) | 0.249 |
| Treatment: Transplant (vs reference) | 0.47 (0.13, 1.74) | 0.259 |
| HLA-C*04 × Treatment: Transplant (interaction) | 1.14 (0.15, 8.89) | 0.898 |

Multivariable Firth Penalized Cox Model

| Predictor | HR (95% CI) | p-value |
| --- | --- | --- |
| HLA-C*04 | 3.34 (0.45, 24.76) | 0.238 |
| Treatment: Transplant (vs reference) | 0.70 (0.08, 5.92) | 0.745 |
| Age (y) | 1.01 (0.93, 1.09) | 0.803 |
| Female (vs Male) | 2.71 (0.44, 16.91) | 0.285 |
| MELD | 1.25 (1.06, 1.48) | **0.010** |
| Cirrhosis | 0.32 (0.07, 1.51) | 0.150 |
| Other malignancies | 6.00 (1.38, 26.04) | **0.017** |
| Largest Tumor Over 3cm | 5.32 (1.27, 22.30) | **0.022** |
| Alcohol Use | 2.11 (0.53, 8.36) | 0.288 |
| HLA-C*09 | 3.44 (0.64, 18.36) | 0.148 |
| HLA-DR*11 | 4.70 (0.65, 34.05) | 0.125 |
| HLA-DQ*07 | 0.57 (0.13, 2.57) | 0.461 |
| HLA-C*04 × Treatment: Transplant (interaction) | 1.49 (0.13, 17.47) | 0.749 |
